# Lowering DVT-Related ED Visits via a Primary Care POCUS Pathway

**DOI:** 10.24908/pocusj.v11i01.20246

**Published:** 2026-04-22

**Authors:** William Hui, Jo-Anne Suffoletto, Steven Lin

**Affiliations:** Division of Primary Care and Population Health, Department of Medicine, Stanford University School of Medicine, Stanford, CA, USA

**Keywords:** deep vein thrombosis, Primary Care, Quality Improvement, Point-of-care ultrasound, POCUS

## Abstract

**Background:**

Lower extremity deep vein thrombosis (DVT) requires prompt diagnosis to prevent pulmonary embolism (PE). DVT point of care ultrasound (POCUS) is shown to be effective in emergency department (ED) settings, though few studies have demonstrated impact in primary care settings. This quality improvement (QI) project aimed to establish a primary care DVT POCUS pathway to reduce ED visits.

**Methods:**

From June 2024 to August 2025, a POCUS fellowship-trained physician at an academic family medicine clinic received referrals from other primary care clinicians for suspected DVTs and performed POCUS evaluations and DVT management. Patient outcomes and clinician feedback were collected via chart review and surveys to assess the project's feasibility and acceptability.

**Results:**

Eighty patients were evaluated, with a positive DVT rate of 5% (4/80). The pathway potentially avoided ED visits for 46% of patients (37/80), translating to an estimated cost savings of $85,100. With a 98% (43/44) response rate, most referring clinicians strongly agreed that the DVT pathway improved patient safety and access, reduced their cognitive burden, and reduced ED referrals.

**Conclusions:**

The DVT POCUS pathway effectively managed patients with suspected DVT in the primary care setting, demonstrating feasibility and acceptability with clinicians, as well as potential cost savings. This model warrants further research as a framework for integrating DVT POCUS into primary care, enhancing diagnostic capabilities and patient care, while alleviating pressures on EDs.

## Introduction

Deep vein thrombosis (DVT) has an annual incidence of 5 per 10,000 people and requires prompt diagnosis to prevent pulmonary embolism (PE), a complication that develops in up to 50% of untreated cases with a mortality rate up to 30% [1,2]. Venous thromboembolism (VTE) costs the United States healthcare system an estimated $5-10 billion annually [3,4].

DVT point of care ultrasound (POCUS) has been effectively utilized in the emergency department (ED) and hospital settings, particularly after hours when radiology services are unavailable, leading to faster patient disposition and reduced time to diagnosis [5–7]. Incorporating DVT POCUS into clinical protocols can result in a 27% reduction in imaging and 67% reduction in D-dimer testing [5]. The proximal DVT POCUS examination demonstrates 90% sensitivity and 97–99% specificity [8–11]. Combining POCUS with risk stratification allows clinicians to diagnose over 80% of DVTs independently [12].

Although DVT POCUS can be effective in ED settings, there are few studies demonstrating its impact in primary care. At our institution from September 2024 to August 2025, urgent outpatient radiology ultrasound had a mean wait time of 2.2 days, extending to 3.7 days for Thursday and Friday orders. In our ED during the same time period, 85 low acuity ED visits were made for urgent DVT ultrasound, resulting in 6 positive DVT and 1 positive PE, and were subsequently discharged home. This project was initiated after an internal chart review revealed low-risk DVT assessments in the ED, likely due to limited outpatient radiology availability during weekday hours. Value-based care is a healthcare delivery model that reimburses providers based on patient health outcomes, quality of care, and efficiency, rather than volume of services rendered. As part of our institution's value-based care initiative, we aimed to establish a DVT POCUS pathway in primary care with the hypothesis that it could provide timely diagnostics, be feasible to use by referring clinicians, and decrease DVT-related ED visits and costs.

## Methods

### Pilot Intervention

**Figure 1.**
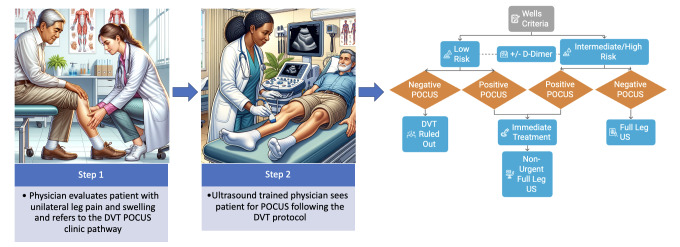
Primary Care DVT POCUS Pathway.

This QI project was conducted at a single family medicine clinic within a large academic medical center in Palo Alto, California, USA, from June 2024 to August 2025. The pathway employed an ultrasound-fellowship trained physician for urgent DVT evaluations seen within 2 days on average (Figure 1). Primary care clinicians referred patients via staff messaging to the ultrasound-fellowship-trained physician. This physician managed scheduling and conducted evaluation, POCUS, and management in the same visit. The pilot was advertised through meetings and emails.

POCUS examination: Scans were performed using a Sonosite Xporte (Fujifilm Sonosite, Bothell, WA, USA) with curvilinear and/or linear probes. The 2-zone POCUS DVT compression ultrasound was utilized [13]. First, the patient was positioned supine with the affected leg externally rotated, in the frog-leg position. The common femoral vein (CFV) and femoral vein (FV) were evaluated from the inguinal ligament to the distal thigh, including the junctions of the great saphenous vein (GSV)-CFV, and the FV and deep femoral vein (DFV). Then, the patient was positioned in the lateral decubitus or standing position to evaluate the popliteal vein and its trifurcation. The compression ultrasound was complete if there was adequate compression of the vein at every 1 cm. Indeterminate studies prompted a full leg radiology ultrasound evaluation.

### Patient selection

Patients were referred to the DVT POCUS pathway by primary care clinicians. Inclusion criteria were unilateral leg pain and swelling. Exclusion criteria included recurrent DVT and signs suggestive of a PE.

### Diagnostic algorithm

Using Wells' criteria, patients were categorized into low (<2) or intermediate/high (≥2) pre-test probability. A D-dimer test was used at the clinician's discretion. DVT was excluded in low risk with a negative POCUS. In moderate/high risk, a negative POCUS was followed by a repeat radiology ultrasound in one week to rule out proximal extension of a distal DVT [9,14–16]. Patients with positive DVT POCUS received immediate treatment, with a non-urgent radiology ultrasound ordered [17] (Figure 1).

### Data collection

Data were collected prospectively, including date of exam request and visit, history, Wells' criteria, radiology, lab results, presumed diagnosis, and treatment. Follow-up data were collected at 3 months to check for VTE diagnoses after a negative DVT POCUS scan. A 5-point Likert scale survey was sent to all referring clinicians to assess attitudes towards the new pathway.

## Results

### Patient Outcomes

Eighty patients (Table 1) were evaluated in the POCUS DVT pathway. They had a mean wait time of 2.1 days, comparable to our institution's average of 2.2 days for urgent radiology DVT studies. Two patients were diagnosed with a positive proximal DVT.

**Table 1.** Patient Characteristics. DVT, deep vein thrombosis; RN, registered nurse; VTE, venous thromboembolism. *Positive DVT includes both proximal and distal DVTs.

Baseline Patient Characteristics	All patients (n = 80)	Positive DVT (n = 4)*	Negative DVT (n = 76)
Mean Age (years)	68.5	66.3	68.6
Male	34	4	30
Female	46	0	46
Previous VTE	13	2	11
Active Cancer	9	2	7
Recent Surgery	5	1	4
Immobilization	11	1	10
Well's criteria low (<2)	62	0	62
Wells' criteria moderate/high (≥2)	18	4	14
Referred from RN	13	1	12

Additionally, two patients were found to have a DVT on follow-up imaging within 1 week. The first patient had a proximal DVT identified as a distal popliteal DVT extending from the calf veins at 1 week after the initial visit. This suggested that a DVT may have extended from a distal to a proximal DVT within the 2 days between the POCUS and radiology scan, or suggested that it was a falsely negative POCUS proximal DVT scan. The second patient had a distal DVT identified on follow-up 1 week after the initial visit, which resolved without treatment.

**Figure 2.**
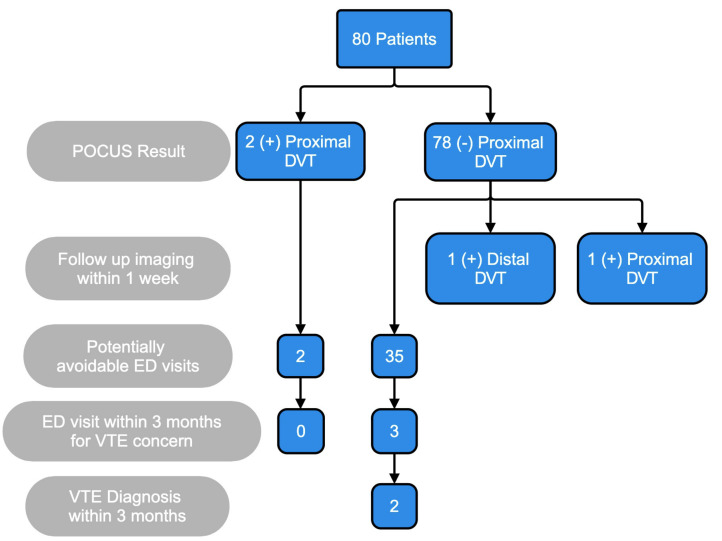
Consort style flowchart showing DVT POCUS results, follow up imaging results, potentially avoidable ED visits, ED visits, and VTE diagnosis within 3 months.

After the initial evaluation, two additional patients had VTE diagnosed within 3 months. One patient with active cancer developed a proximal DVT that was likely due to their ongoing clotting risk factors. The second patient had a distal DVT detected on radiology ultrasound due to persistent leg symptoms (Figure 2).

### Estimated Financial Impact

From the study, 46% (37/80) of cases were potentially avoidable ED visits (defined as requesting an exam on Thursday/Friday). This saved an average ED level 4 or 5 cost of $2,300 per patient, or a total of $85,100, along with the indirect cost savings of freeing up an ED slot for a higher level of care.

The outpatient POCUS pathway costs approximately $239.69 per case (Using the CMS Medicare Physician fee schedule for 2025: $114.51 (93971 limited DVT POCUS) + $125.18 (99214 office visit)).

### Referring Provider Perspectives

**Figure 3.**
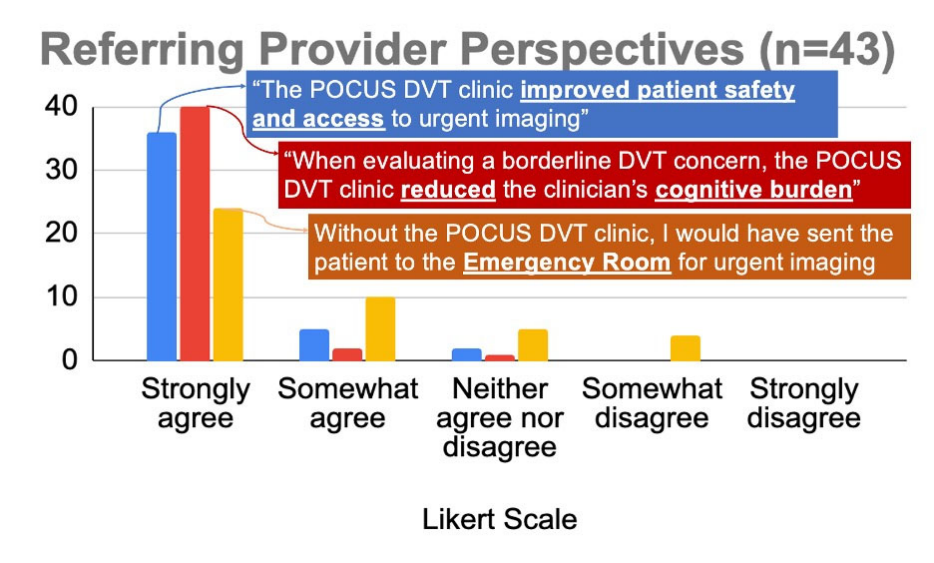
Referring Provider Perspectives towards the DVT POCUS pathway.

A survey was sent to all 44 referring clinicians, with a response rate of 98% (43/44). Most providers indicated that the POCUS DVT clinic improved patient safety and access, reduced cognitive burden, and avoided unnecessary ED referrals (Figure 3).

## Discussion

To our knowledge, this is the first QI study demonstrating the impact of a referral-based DVT POCUS pathway in outpatient primary care. The pathway effectively managed patients with suspected DVT, demonstrating feasibility and acceptability with clinicians and potential cost savings.

The establishment of the DVT POCUS pathway had slow initial uptake, but through advertising and collaborations with registered nurses, the service has spread organically, reaching a rate of about one referral per week. Yearly advertisement is planned to sustain this project. There may be additional opportunities to scale to the broader ambulatory setting, including subspecialty care. However, despite its utility, clinician referrals remain low, possibly due to insufficient provider education on the benefits and limitations of POCUS. DVT assessment is high-stakes, and clinicians may be hesitant to refer for a limited POCUS study over traditional methods. In addition, the average wait time for the POCUS pathway and our institution's urgent radiology DVT orders were similar at about 2 days. The POCUS pathway wait time is related to the availability of clinic slot scheduling. It is worth pointing out that the POCUS pathway is particularly useful towards the end of the week, as wait times for radiology ultrasound lengthen to 3.7 days on Thursday and Friday, as no available outpatient radiology services are available over the weekend. It is helpful to have an adjunct POCUS service to help offload radiology pressures and ED visits.

## Limitations

This project was conducted by a single US-fellowship-trained family physician, limiting generalizability. Referrals are manually scheduled, which can be time intensive. Future steps include involving the medical assistant scheduling team and exploring integration into an automatic electronic medical record referral.

Additionally, the chart review utilized the academic institution's electronic medical record (Epic Systems, Verona, Wisconsin, USA). Therefore, VTE cases occurring outside of this system may have been missed in the retrospective review.

In this study, a DVT prevalence of 5% (4/80) was seen, as compared to the 19% prevalence rate for outpatient DVTs [18]. This may suggest an inadequate sample size. However, the majority of the patients were low risk, which matches the prevalence rate for low pretest probability of 3.5-8.1% [9].

## Conclusions

Utilizing POCUS in the primary care setting effectively triages patients with suspected DVT, serving as an adjunct to traditional radiology services.

Though POCUS is usually an examination done at the time of initial visit, our pilot demonstrated that a referral-based DVT POCUS pathway was safe and effective, deemed feasible and acceptable by clinicians, and generated potential cost savings. This model warrants further research as a framework for integrating DVT POCUS into primary care, enhancing diagnostic capabilities and patient care, while alleviating pressures on EDs.

## References

[1] Fernandez MM, Hogue S, Preblick R, Kwong WJ. Review of the cost of venous thromboembolism. Clin Outcomes Res CEOR. 2015;7:451–462. doi:10.2147/CEOR.S85635 PMC4559246 26355805

[2] Fowkes FJI, Price JF, Fowkes FGR. Incidence of diagnosed deep vein thrombosis in the general population: systematic review. Eur J Vasc Endovasc Surg Off J Eur Soc Vasc Surg. 2003;25(1):1–5. doi:10.1053/ejvs.2002.1778 12525804

[3] Grosse SD. Incidence-based cost estimates require population-based incidence data. A critique of Mahan et al. Thromb Haemost. 2012;107(1):192–193; author reply 194-195. doi:10.1160/TH11-09-0666 22159589

[4] Grosse SD, Nelson RE, Nyarko KA, Richardson LC, Raskob GE. The economic burden of incident venous thromboembolism in the United States: A review of estimated attributable healthcare costs. Thromb Res. 2016;137:3–10. doi:10.1016/j.thromres.2015.11.033 26654719 PMC4706477

[5] Poley RA, Newbigging JL, Sivilotti MLA. Estimated Effect of an Integrated Approach to Suspected Deep Venous Thrombosis Using Limited-compression Ultrasound. Acad Emerg Med. 2014;21(9):971–980. doi:10.1111/acem.12459 25269577

[6] American College of Emergency Physicians. Emergency ultrasound guidelines. Ann Emerg Med. 2009;53(4):550–570. doi:10.1016/j.annemergmed.2008.12.013 19303521

[7] Fischer EA, Kinnear B, Sall D, Kelleher M, Sanchez O, Mathews B, Schnobrich D, Olson A. Hospitalist-Operated Compression Ultrasonography: a Point-of-Care Ultrasound Study (HOCUS-POCUS). J Gen Intern Med. 2019;34(10):2062–2067. doi:10.1007/s11606-019-05120-5 31388904 PMC6816719

[8] Hercz D, Mechanic OJ, Varella M, Fajardo F, Levine RL. Ultrasound Performed by Emergency Physicians for Deep Vein Thrombosis: A Systematic Review. West J Emerg Med. 2024;25(2):282–290. doi:10.5811/westjem.18125 38596931 PMC11000565

[9] Lim W, Le Gal G, Bates SM, Righini M, Haramati LB, Lang E, Kline JA, Chasteen S, Snyder M, Patel P, Bhatt M, Patel P, Braun C, Begum H, Wiercioch W, Schünemann HJ, Mustafa RA. American Society of Hematology 2018 guidelines for management of venous thromboembolism: diagnosis of venous thromboembolism. Blood Adv. 2018;2(22):3226–3256. doi:10.1182/bloodadvances.2018024828 30482764 PMC6258916

[10] Varrias D, Palaiodimos L, Balasubramanian P, Barrera CA, Nauka P, Melainis AA, Zamora C, Zavras P, Napolitano M, Gulani P, Ntaios G, Faillace RT, Galen B. The Use of Point-of-Care Ultrasound (POCUS) in the Diagnosis of Deep Vein Thrombosis. J Clin Med. 2021;10(17):3903. doi:10.3390/jcm10173903 34501350 PMC8432124

[11] Mumoli N, Vitale J, Giorgi-Pierfranceschi M, Sabatini S, Tulino R, Cei M, Bucherini E, Bova C, Mastroiacovo D, Camaiti A, Palmiero G, Puccetti L, Dentali F. General Practitioner–Performed Compression Ultrasonography for Diagnosis of Deep Vein Thrombosis of the Leg: A Multicenter, Prospective Cohort Study. Ann Fam Med. 2017;15(6):535–539. doi:10.1370/afm.2109 29133492 PMC5683865

[12] Cherkaoui M, Al-Attabi M, Salimi S, Cherkaoui B, Forberg JL. Proximal venous ultrasound with risk stratification safely excludes deep venous thrombosis in emergency department routine care: an observational study. Scand J Trauma Resusc Emerg Med. 2025;33(1):85. doi:10.1186/s13049-025-01382-7 40369661 PMC12077004

[13] Barrosse-Antle ME, Patel KH, Kramer JA, Baston CM. Point-of-Care Ultrasound for Bedside Diagnosis of Lower Extremity DVT. Chest. 2021;160(5):1853–1863. doi:10.1016/j.chest.2021.07.010 34270964

[14] Bates SM, Jaeschke R, Stevens SM, Goodacre S, Wells PS, Stevenson MD, Kearon C, Schunemann HJ, Crowther M, Pauker SG, Makdissi R, Guyatt GH. Diagnosis of DVT: Antithrombotic Therapy and Prevention of Thrombosis, 9th ed: American College of Chest Physicians Evidence-Based Clinical Practice Guidelines. CHEST. 2012;141(2):e351S–e418S. doi:10.1378/chest.11-2299 22315267 PMC3278048

[15] Mazzolai L, Aboyans V, Ageno W, Agnelli G, Alatri A, Bauersachs R, Brekelmans MPA, Büller HR, Elias A, Farge D, Konstantinides S, Palareti G, Prandoni P, Righini M, Torbicki A, Vlachopoulos C, Brodmann M. Diagnosis and management of acute deep vein thrombosis: a joint consensus document from the European Society of Cardiology working groups of aorta and peripheral vascular diseases and pulmonary circulation and right ventricular function. Eur Heart J. 2018;39(47):4208–4218. doi:10.1093/eurheartj/ehx003 28329262

[16] Kraaijpoel N, Carrier M, Le Gal G, McInnes MDF, Salameh JP, McGrath TA, van Es N, Moher D, Büller HR, Bossuyt PM, Leeflang MMG. Diagnostic accuracy of three ultrasonography strategies for deep vein thrombosis of the lower extremity: A systematic review and meta-analysis. PloS One. 2020;15(2):e0228788. doi:10.1371/journal.pone.0228788 32045437 PMC7012434

[17] Needleman L, Cronan JJ, Lilly MP, Merli GJ, Adhikari S, Hertzberg BS, DeJong MR, Streiff MB, Meissner MH. Ultrasound for Lower Extremity Deep Venous Thrombosis. Circulation. 2018;137(14):1505–1515. doi:10.1161/CIRCULATIONAHA.117.030687 29610129

[18] Wells PS, Owen C, Doucette S, Fergusson D, Tran H. Does This Patient Have Deep Vein Thrombosis? JAMA. 2006;295(2):199–207. doi:10.1001/jama.295.2.199 16403932

